# Hydrogen-bond network and pH sensitivity in human transthyretin

**DOI:** 10.1107/S090904951302075X

**Published:** 2013-09-29

**Authors:** Takeshi Yokoyama, Mineyuki Mizuguchi, Yuko Nabeshima, Katsuhiro Kusaka, Taro Yamada, Takaaki Hosoya, Takashi Ohhara, Kazuo Kurihara, Ichiro Tanaka, Nobuo Niimura

**Affiliations:** a Faculty of Pharmaceutical Sciences, University of Toyama, 2630 Sugitani, Toyama 930-0914, Japan; b Frontier Research Center for Applied Atomic Sciences, Ibaraki University, 162-1 Shirakata, Tokai, Ibaraki 319-1106, Japan; c College of Engineering, Ibaraki University, 4-12-1 Naka-Narusawa, Hitachi, Ibaraki 316-8511, Japan; d Research Center for Neutron Science and Technology, Comprehensive Research Organization for Science and Society, 162-1 Shirakata, Tokai, Ibaraki 319-1106, Japan; e Quantum Beam Science Directorate, Japan Atomic Energy Agency, 2-4 Shirakata, Tokai, Ibaraki 319-1195, Japan

**Keywords:** neutron protein crystallography, transthyretin, amyloidosis, hydrogen-bond network, pH sensitivity

## Abstract

Transthyretin (TTR) is a tetrameric protein. TTR misfolding and aggregation are associated with human amyloid diseases. Dissociation of the TTR tetramer is believed to be the rate-limiting step in the amyloid fibril formation cascade. Low pH is known to promote dissociation into monomer and the formation of amyloid fibrils. In order to reveal the molecular mechanisms underlying pH sensitivity and structural stabilities of TTR, neutron diffraction studies were conducted using the IBARAKI Biological Crystal Diffractometer with the time-of-flight method. Crystals for the neutron diffraction experiments were grown up to 2.5 mm^3^ for four months. The neutron crystal structure solved at 2.0 Å revealed the protonation states of His88 and the detailed hydrogen-bond network depending on the protonation states of His88. This hydrogen-bond network is involved in monomer–monomer and dimer–dimer interactions, suggesting that the double protonation of His88 by acidification breaks the hydrogen-bond network and causes the destabilization of the TTR tetramer. Structural comparison with the X-ray crystal structure at acidic pH identified the three amino acid residues responsible for the pH sensitivity of TTR. Our neutron model provides insights into the molecular stability related to amyloidosis.

## Abstract 2

The neutron crystal structure of human transthyretin is presented.

## 1. Introduction

Amyloidosis refers to a variety of conditions wherein normally soluble proteins become insoluble and are deposited in the extracellular space of various organs or tissues, causing damage. Transthyretin (TTR) is a tetrameric protein and transports hormone thyroxine and retinol A in the blood. TTR misfolding and aggregation are associated with amyloid diseases such as senile systemic amyloidosis, familial amyloid polyneuropathy and familial amyloid cardiomyopathy (Rochet & Lansbury, 2000 ▶; Buxbaum & Tagoe, 2000 ▶; Kelly, 1996 ▶; Benson, 1989 ▶).

**Figure 1:**
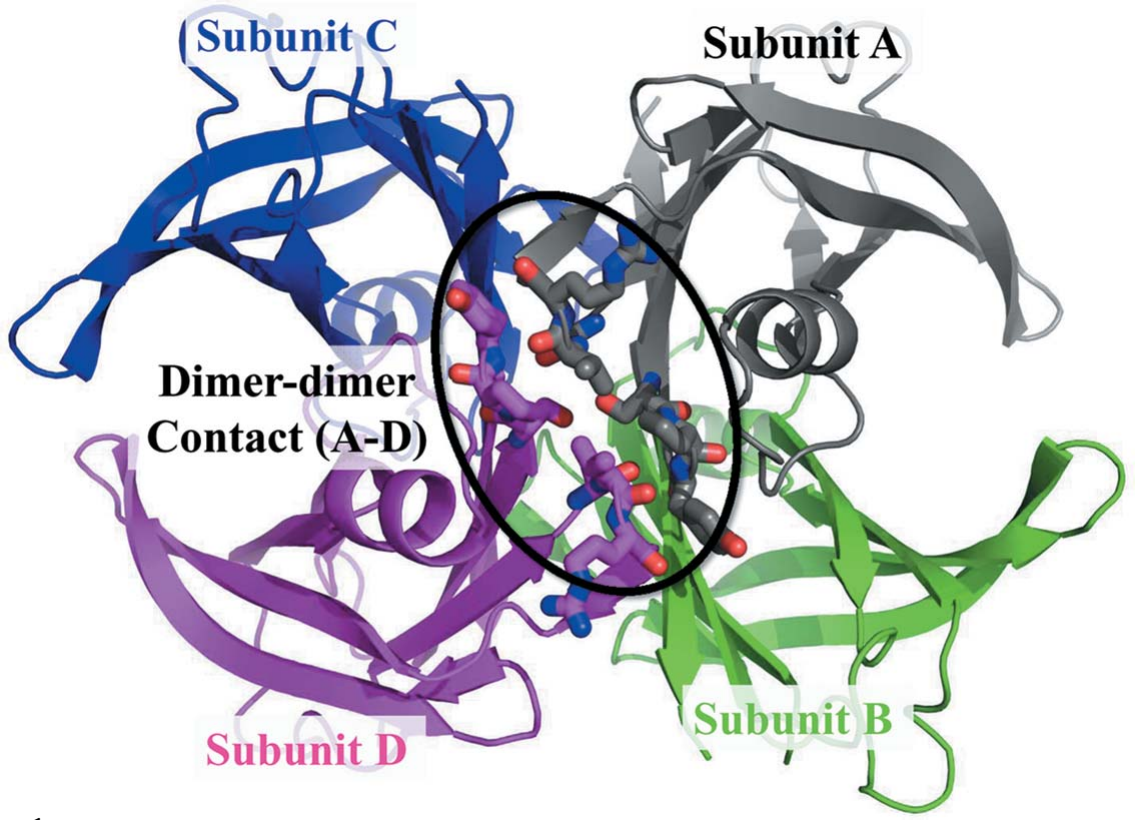
Structure of the TTR tetramer. The dimer–dimer contacts are indicated as dashed circles. Subunit *A* is shown in grey, *B* in green, *C* in blue and *D* in magenta.

TTR is a tetramer of 55 kDa composed of four identical polypeptide chains (subunits *A*–*D*) of 127 amino acid residues (Fig. 1 ▶). Each polypeptide chain forms eight β-strands and one α-helix. The intersubunit contacts are divided roughly into monomer–monomer interactions and dimer–dimer inter­actions. The monomer–monomer interactions are formed between subunits *A* and *B* or *C* and *D*, whereas the dimer–dimer interactions are formed between subunits *A* and *D* or *B* and *C*. These contacts are important for the stability of the TTR tetramer. The mechanism underlying TTR amyloidogenesis in humans is the subject of intense investigation. Rate-limiting dissociation of the tetramer into its component subunits is necessary. The folded subunits must also undergo partial denaturation to produce an amyloidogenic intermediate, a step that is often linked thermodynamically to dissociation. This intermediate then misassembles into numerous morphologies including amorphous aggregates and spherical aggregates, and ultimately into amyloid fibrils (Lashuel *et al.*, 1998 ▶, 1999 ▶; Colon & Kelly, 1992 ▶). While the acidic conditions greatly accelerate the rate-limiting TTR dissociation and the aggregate formation, some small molecules, that bind to TTR, kinetically stabilize TTR and suppress the amyloid fibril formation (Bulawa *et al.*, 2012 ▶; Hurshman *et al.*, 2004 ▶; Liu *et al.*, 2000 ▶; Kelly *et al.*, 1997 ▶; Klabunde *et al.*, 2000 ▶). Recent structural studies have begun to reveal the structural changes by the lowered pH in both wild-type and amyloidogenic mutant TTR. The crystal structure of the I84A amyloidogenic mutant showed notable conformational changes at pH 4.6 compared with that of the I84A structure determined at pH 7.5. In these structures a large conformational change is found at the EF-helix and loop (Pasquato *et al.*, 2007 ▶). Furthermore, the crystal structure of the wild-type TTR determined at pH 4.0 and 3.5 also showed conformational changes in the same region (Palaninathan *et al.*, 2008 ▶). Although many X-ray crystal structures of TTR have been solved so far, the precise molecular mechanisms underlying TTR aggregation remain elusive. The pH-dependent effects in proteins are mainly electrostatic in nature and originate from changes in the protonation states of acidic and basic residues (Yang & Honig, 1993 ▶). To further investigate the structural explanation for the pH-dependent effect of TTR, detailed information on the hydrogen and protonation states is needed. The neutron protein crystallography is preferred as a tool to determine the hydrogen bonding, the protonation states and the hydration of macromolecules, since the neutron-scattering lengths of hydrogen and deuterium are comparable with those of other elements (Niimura & Bau, 2008 ▶). We report here the neutron crystallographic analysis of TTR (Yokoyama *et al.*, 2012 ▶). The neutron crystal structure solved at 2.0 Å provides the protonation states and detailed information about the hydrogen bonds. We discuss the origin of pH sensitivity related to the structural stability of TTR.

## 2. Materials and methods

### 2.1. Protein preparation and crystallization

**Figure 2:**
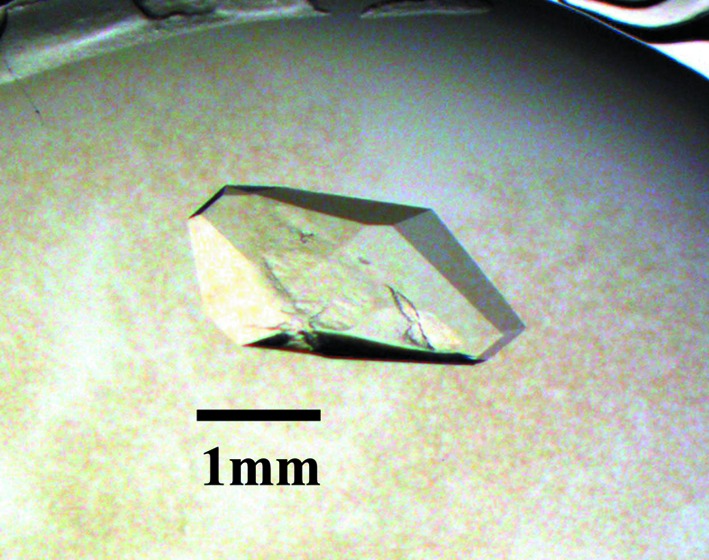
The TTR crystal for the neutron diffraction experiment.

In order to obtain a large crystal suitable for neutron crystallography, an N-terminal truncated TTR lacking 1–11 was expressed (Yokoyama *et al.*, 2012 ▶). The expression and the purification of N-terminal truncated TTR were carried out as previously described (Miyata *et al.*, 2010 ▶). The purified protein was concentrated up to 19 mg ml^−1^ and frozen with liquid nitrogen until use. As N-terminal truncated TTR was likely to crystallize only from the magnesium-ion-containing solutions as a result of the many crystallization screenings, the crystallization screenings were carried out again using protein solution supplemented with 0.2 *M* MgCl_2_. Single crystals were observed using tri-ammonium citrate pH 7.0 as the precipitating agent within a few days. In order to avoid neutron incoherent scattering from H atoms, the crystals for the neutron diffraction experiments were grown using the protein solution exchanged by heavy water and precipitating agents prepared with the heavy water. The large crystal of N-terminal truncated TTR was obtained in a drop containing 1.85 *M* tri-ammonium citrate pD 7.4 and 0.4 *M* MgCl_2_ at 293 K in four months by the sitting-drop vapour-diffusion method (Fig. 2 ▶).

### 2.2. Neutron diffraction experiments and structure refinement

**Figure 3:**
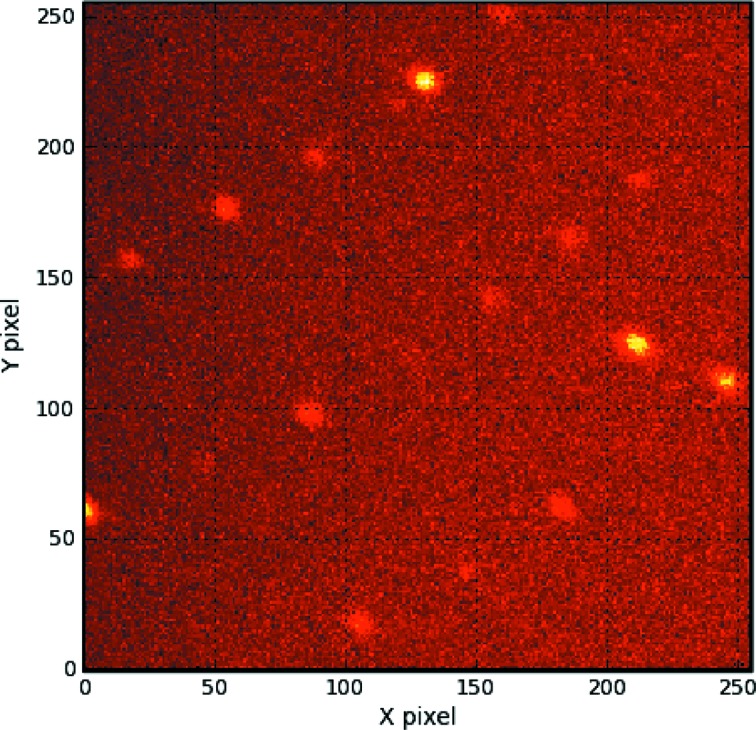
Time-of-flight neutron diffraction image recorded by iBIX. The three-dimensional diffraction data were projected in time-of-flight.

Crystals were mounted in quartz capillaries with the reservoir solution to avoid dryness and then the capillaries were sealed with wax. Time-of-flight neutron diffraction data were collected in the BL-03 iBIX installed at the pulsed neutron source of MLF in J-PARC (Tanaka *et al.*, 2010 ▶). The diffraction data sets were collected at room temperature using 13 detectors placed at 2θ_center_ from 33.0° to 139.4° (Hosoya *et al.*, 2009 ▶). To complete the data, 41 data sets (41 crystal orientations from one crystal) were collected using a wavelength range from 2.7 Å to 6.7 Å with a crystal-to-detector distance of 490 mm. The exposure times were 22 h for each set at 120 kW J-PARC accelerator power and 12 h at 220 kW. The diffraction peaks were observed distinctly (Fig. 3 ▶). The collected data were indexed, integrated and scaled with *STARGazer* which was developed to process iBIX time-of-flight diffraction data (Ohhara *et al.*, 2009 ▶). The X-ray crystal structure of TTR at room temperature was used as the initial model (Protein Data Bank ID: 3u2i). The structure was refined using *PHENIX-REFINE* for neutron structure refinement with several stepwise cycles of manual model building using *COOT* (Adams *et al.*, 2011 ▶; Emsley & Cowtan, 2004 ▶). The data collection and refinement statistics are listed in Table 1 ▶.

## 3. Results and discussion

### 3.1. Hydrogen-bond network and pH sensitivity

**Figure 4:**
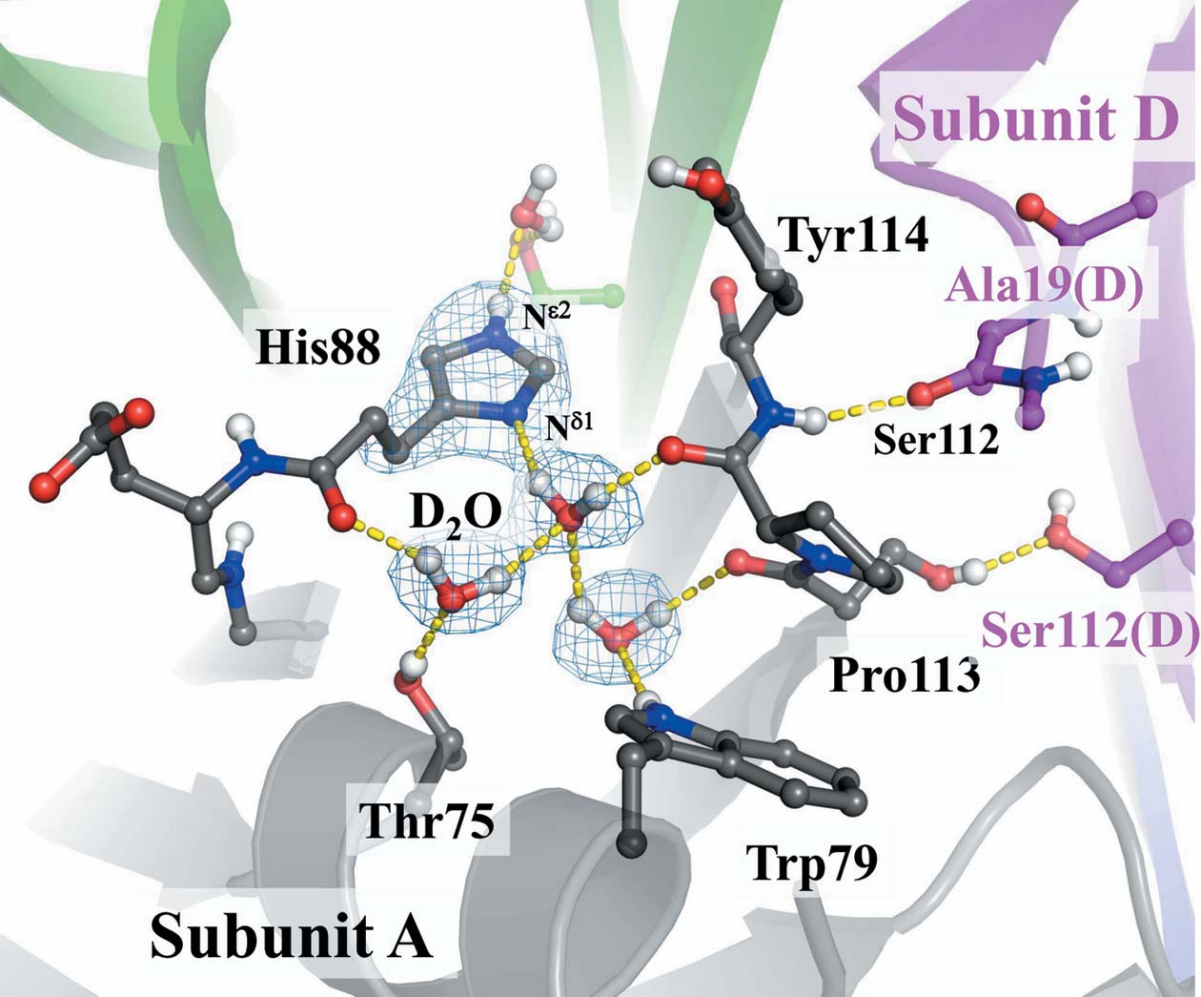
The hydrogen-bond network consisted of His88 and water molecules. The |*F*_o_| − |*F*_c_| difference neutron scattering length density map was calculated omitting His88 and water molecules (contoured at 2.5σ). The hydrogen bonds are indicated as dashed yellow lines. The residue names of subunit *A* are shown in grey and those of *D* are in magenta. Unexchangeable H atoms are not displayed.

The neutron structure of TTR was identical to the X-ray structure of TTR with a root-mean-square deviation of 0.34 Å between the C^α^ atoms of the two structures. First, the protonation states of the histidine residues were determined based on the Fourier peaks at the positions of hydrogen (deuterium) atoms of the difference Fourier map omitting each histidine residue. The Fourier density showed that His31 was doubly protonated, whereas His56, His88 and His90 were singly protonated. Furthermore, the orientations of the water molecules were determined by the same method. Fourteen out of 55 water molecules observed in the asymmetric unit were identified as complete water (D_2_O but neither DO nor O). Among the four histidine residues, the protonation state of His88 is very interesting. The unprotonated N^δ1^ atom of His88 accepted a hydrogen bond from the water molecule (Fig. 4 ▶). This hydrogen bond was involved in a large hydrogen-bond network consisting of Thr75, Trp79, His88, Ser112, Pro113, Thr118(B) and four water molecules. As this hydrogen-bond network was made up of ten hydrogen bonds, it is important for the structural stability of TTR. It is suggested that the double protonation of His88 may break this hydrogen-bond network and destabilize the TTR monomer structure. This network is also involved in the dimer–dimer interaction, which is important for the tetramer formation of TTR (Fig. 4 ▶). This interaction includes the two hydrogen bonds formed between Ser112 of subunit *A* and Ser112 of *D* and between Tyr114 of subunit *A* and Ala19 of *D*. These hydrogen bonds appeared to be important in light of the fact that Ser112Ile and Tyr114His mutants are amyloidogenic variants (Murakami *et al.*, 1994 ▶; Shinohara *et al.*, 2003 ▶). This dimer–dimer arrangement is stabilized by the hydrogen-bond network of His88 stabilizing the GH-loop (Pro113 and Tyr114) (Fig. 4 ▶). As Ser112 and Pro113 are members of the hydrogen-bond network, the full protonation of His88 by acidic condition probably breaks this hydrogen-bond network and destabilizes the tetramer. In order to determine the residues responsible for the pH sensitivity of TTR, the neutron structure of TTR was compared with the X-ray structure at pH 4.0 (Palaninathan *et al.*, 2008 ▶). The conformational changes of Asp74, His88 and Glu89 were observed by structural comparison. Asp74 forms a hydrogen bond with Ser77 at neutral pH, whereas it forms a hydrogen bond with a water molecule on the molecular surface at pH 4.0. His88 is involved in the large hydrogen-bond network at neutral pH but swings away into the solvent without any hydrogen bonds with the water molecules at pH 4.0. Glu89 forms a salt bridge with Lys76 at neutral pH, whereas the salt bridge is broken at pH 4.0. These results suggest that Asp74, His88 and Glu89 are mainly responsible for the pH sensitivity of TTR. Among these residues, His88 involves the large hydrogen-bond network composed of ten hydrogen bonds. Therefore, His88 is likely predominant in pH sensitivity.

### 3.2. CH⋯O hydrogen bond

Careful inspection of the H atoms affords some clues to understanding the CH⋯O weak hydrogen bonds. The close CH⋯O contacts are thought to play an important role in the stabilization and function of biological molecules. CH⋯O contacts are increasingly being accepted as genuine hydrogen bonds (Desiraju & Steiner, 1999 ▶; Wahl & Sundaralingam, 1997 ▶). According to the energies calculated by Jiang & Lai (2002 ▶), the CH⋯O hydrogen bond has a binding strength of −1.9 kJ mol^−1^ and an optimum C⋯O distance of 3.3 Å, whereas the conventional hydrogen bond has a binding energy of −5.5 kJ mol^−1^ and an optimum O⋯O (N) distance of 2.8 Å. The list of hydrogen bonds and possible CH⋯O hydrogen bonds formed in the dimer–dimer contact (subunits *A* and *D*) are summarized (Table 2 ▶). Not only three hydrogen bonds but also eight CH⋯O hydrogen bonds are formed in this region. A simple calculation of the energies underscores the importance of CH⋯O hydrogen bonds, because the energy sum of these bonds is comparable with that of conventional hydrogen bonds and is not negligible. At this interface, Tyr114 seems to be a major contributor to the intersubunit contact. The substitution of Tyr114 with His, which is known as an amyloidogenic variant, perturbed the stability of the quaternary structure. These results suggest that CH⋯O hydrogen bonds may play an important role in stabilizing the quaternary structure of TTR.

## 4. Conclusions

Large TTR crystals with a volume of 2.5 mm^3^ were obtained and the neutron crystal structure was solved at 2.0 Å resolution using iBIX. The neutron structure revealed that the protonation state of His88 is closely related to tetramer stability. The mechanisms underlying accelerated amyloid fibril formation by acidic conditions were structurally explained by neutron protein crystallography. Although it is difficult to obtain crystals large enough for neutron diffraction experiments, these results comfirmed the usefulness of neutron protein crystallography.

## Figures and Tables

**Table 1:** Statistics on the data collection and refinement Numbers in parentheses refer to the highest-resolution shell.

Crystal data	
Resolution range (Å)	12.1–2.0 (2.07–2.00)
Space group	*P*2_1_2_1_2
Unit cell (Å)	*a* = 44.3, *b* = 86.4, *c* = 66.7
Unique reflections	15307 (2080)
*R*_sym_ (%)†	19.1 (30.6)
Completeness (%)	86.4 (72.5)
*I*/σ	4.3 (1.5)
Redundancy	2.6 (1.7)

Refinement data	
*R*_factor_ (%)‡	23.4
*R*_free_ (%)§	27.2
RMSD bonds (Å)	0.010
RMSD angles (°)	1.207

† *R*_sym_ = Σ_*hkl*_Σ_*i*_|*I*_*i*_(*hkl*) − *I*(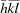)|/Σ_*hkl*_Σ_*i*_ *I*_*i*_(*hkl*).

‡ *R*_factor_ = Σ|*F*_o_| − |*F*_c_|/|*F*_o_|, where *F*_o_ and *F*_c_ are the observed and calculated structure factor amplitudes, respectively.

§ *R*_free_ was calculated with 5% of the data excluded from the refinement.

**Table 2:** List of hydrogen bonds and possible CH⋯O hydrogen bonds formed between subunits *A* and *D*

Acceptor	Donor	Distance (Å)
Hydrogen bonds		O⋯O, N (O⋯*D*)
A19(*A*)–O	Y114(*D*)–*D*	3.0 (2.2)
S112(*A*)–O^γ^	S112(*D*)–*D* ^γ^	2.5 (1.9)
A19(*D*)–O	Y114(*A*)–*D*	3.0 (2.2)

CH⋯O hydrogen bonds		O⋯C (O⋯H)
A19(*A*)–O	Y114(*D*)–H^β2^	3.4 (2.6)
V20(*A*)–O	P113(*D*)–H^β2^	3.6 (2.8)
V20(*A*)–O	Y114(*D*)–H^δ1^	3.4 (2.8)
A19(*A*)–O	S112(*D*)–H^β3^	3.6 (2.9)
A19(*D*)–O	S112(*A*)–H^β3^	3.6 (2.9)
V20(*D*)–O	P113(*A*)–H^β2^	3.6 (2.7)
A19(*D*)–O	Y114(*A*)–H^β2^	3.4 (2.6)
V20(*D*)–O	Y114(*A*)–H^δ1^	3.4 (2.8)
